# Supplementary material for: IPA-3: An Inhibitor of Diadenylate Cyclase of Streptococcus suis with Potent Antimicrobial Activity
Source: Antibiotics (Basel). 2022 Mar 21;11(3):418. doi: 10.3390/antibiotics11030418 (PMC8944544; doi:10.3390/antibiotics11030418)
Supplement: Supplementary file 1 [file antibiotics-11-00418-s001.zip › antibiotics-1622071-supple.pdf]

**Supplementary Table S1. Bacterial strains and plasmids used in this study**

| Name | Description | Reference or source |
|---|---|---|
| Strains | | |
| *S. suis* SC19 | *S. suis* serotype 2 | [1] |
| *S. suis* SS2041 | Multiple-drug resistant strain | [2] |
| *E. coli* ATCC25922 | *E. coli* standard strain | Laboratory stock |
| *S. aureus* ATCC29213 | *S. aureus* standard strain | Laboratory stock |
| *S. aureus* 1213M4A | Multiple-drug resistant strain | Laboratory stock |
| *E. rhusiopathiae 13013* | Multiple-drug resistant strain | [3] |
| *S. mutants* ATCC25175 | *S. mutans* standard strain | Laboratory stock |
| *B. subtilis* WB800N | Derivative of *B. subtilis* 168 | [4] |
| Plasmids | | |
| pET28a-*dacAcyto* | Plasmid for the expression of ssDacA, $Kan^r$ | This study |

**Supplementary Table S2. Primers used in this study**

| Strains | Sequence (5'-3') |
|---|---|
| $ssDacA_{cyto}$-F | TAAGAAGGAGATATACCATGGGCGCTATGCTTGAAAAACTC |
| $ssDacA_{cyto}$-R | GTGGTGGTGGTGGTGCTCGAGTTCATGTTTGCCACCTCC |


1. Li, W.; Liu, L.; Qiu, D.; Chen, H.; Zhou, R. Identification of *Streptococcus suis* serotype 2 genes preferentially expressed in the natural host. Int J Med Microbiol **2010**, *300*, 482-488, doi:10.1016/j.ijmm.2010.04.018.
2. Zou, G.; Zhou, J.; Xiao, R.; Zhang, L.; Cheng, Y.; Jin, H.; Li, L.; Zhang, L.; Wu, B.; Qian, P.; et al. Effects of Environmental and Management-Associated Factors on Prevalence and Diversity of *Streptococcus suis* in Clinically Healthy Pig Herds in China and the United Kingdom. Appl Environ Microbiol **2018**, *84*, doi:10.1128/aem.02590-17.
3. Ding, Y.; Zhu, D.; Zhang, J.; Yang, L.; Wang, X.; Chen, H.; Tan, C. Virulence determinants, antimicrobial susceptibility, and molecular profiles of *Erysipelothrix rhusiopathiae* strains isolated from China. Emerg Microbes Infect **2015**, *4*, e69, doi:10.1038/emi.2015.69.
4. Jeong, H.; Jeong, D.E.; Park, S.H.; Kim, S.J.; Choi, S.K. Complete Genome Sequence of *Bacillus subtilis* Strain WB800N, an Extracellular Protease-Deficient Derivative of Strain 168. Microbiol Resour Announc **2018**, *7*, doi:10.1128/mra.01380-18.
